# The Death of King Edward VII

**Published:** 1910-05-28

**Authors:** 


### Institutional Notes and News.

### THE DEATH OF KING EDWARD VII.

**Queen's Hospital for Children.**

One of the many instances in which the hospitals have had to mourn the death of the late King is to be recorded in the following account of a Royal promise to the Queen's Hospital for Children. A week before King Edward's death the committee received authority to announce that his Majesty and Queen Alexandra would visit the institution during the present summer. No institution, therefore, will have a keener regret, a more personal loss to record than the Queen's Hospital has at the present time. A circular was printed, saying that the Queen had graciously consented to receive purses in aid of the hospital on that occasion and appealing for purses of £10 10s. and upwards as well as for other donations and new annual subscriptions. We may remind our readers that it was by special permission of Queen Alexandra that the Queen's Hospital, established in 1867 as the North-Eastern Hospital for Children, took its present name. The hospital has been passing through a financial crisis, which a visit from King Edward and the Queen-Mother would have done immeasurable service to overcome. It is to be hoped that the people of Hackney, and a wider public still, will understand that the best proof of their loyalty to the late King's memory, and of their friendship for the hospital itself, would be found in making special efforts to do in part what the King's visit alone would have done wholly in filling the hospital's exchequer at this time of need.

**Lord Rosebery and King George.**

A special Court of Governors of the Royal Scottish Hospital, which was held last week in Crane Court to express grief at his late Majesty's death, and loyalty to his Majesty King George V., was fortunate in securing the Earl of Rosebery as chief speaker. After alluding to the war which marked King Edward's accession, and the peace which through his efforts crowned the end of his reign, his lordship said : "We are thinking of two Kings to-day, the King to whom I am moving the address and the King whom we have lost. We shall not readily turn our thoughts from our dead King to our living King, and yet we cannot but be full to-day of that figure which is called to fill the greatest Throne in the world under circumstances so tragic and so unexpected. We may look, I think, with real hope, real confidence, to the reign of our new King, should he be spared to us. He has led the life of a sailor, and we in Great Britain all love sailors. He has led a pure, healthy, and abstemious life; he is a good husband and a good father. He will exhibit on the Throne domestic virtues which are dear to this country. He has explored every region of the Empire over which he is called to rule more than any other Sovereign in the long line of his predecessors. He knows what he has to govern, and at home he has spared no pains to make himself acquainted with every phase of our political life. Only a few days ago Lord Balfour, Lord Kinnaird, and I saw him sitting in the House of Lords, as he was always sitting, as a peer among his peers, listening patiently and vigilantly to the debates in that House. Into the Gallery of the House of Commons he has also gone whenever any interesting debate has been taking place, in the attempt to study in the popular Chamber all the varying politics of the day. He has spared no pains to fit himself for the Throne, and if an honest and earnest endeavour to do his duty be any guarantee of success, that I am sure we have in our new King. There was a formula in France, 'The King is dead; long live the King!' We do not adopt that formula in England. The break is too sudden for our minds, but this at least we can say with all our hearts : 'God bless and preserve the memory of King Edward; God bless and prolong the reign of King George.'" (Hear, hear.)—Lord Balfour of Burleigh, in seconding the motion, reminded the meeting that King George, as a member of the Royal Commission on the Food Supply in Time of War, had attended every meeting.—Lord Rosebery, replying to a vote of thanks, said he regarded it as one of the greatest honours of his life to have been privileged to succeed our late King as President of the Royal Scottish Corporation.

**Leicester Infirmary.**

At the meeting of the Board of Governors of the Leicester Infirmary, held on Wednesday, the following resolution was passed in silence. Moved by Sir Edward Wood (chairman of the Board), seconded by Sir Arthur Hazlerigg (vice-chairman), and resolved : "That the Board of Governors of the Leicester Infirmary record their profound grief at the severe blow which has fallen upon this Kingdom and Empire by the death of its beloved Sovereign, King Edward VII., whose reign will always rank in the annals of history as that of a great and wise monarch whose chief aim was to promote the true happiness and welfare of his people, and to preserve, strengthen, and maintain peace and goodwill throughout the world. That the Board acknowledge with deep gratitude the immense impetus which has been given during his Majesty's reign to the work of hospitals, charities, and all charitable effort by the constant and special solicitude which his Majesty always manifested in the cause of the sick and suffering, with which work his memory will always be associated. That the Board dutifully tender to her Majesty the Queen Mother, to their Majesties George V. and Queen Mary, and to all the members of the Royal Family the expression of their deep and heartfelt sympathy, and to his Majesty King George V. their congratulations on his accession to the Throne, the assurance of their loyalty and attachment to his Majesty's Throne and person, and the earnest prayer that his Majesty's reign may be long and distinguished and productive of happiness to his Majesty and prosperity for the British Empire."

**Worcester General Infirmary.**

The proceedings at the meeting of the Executive Committee of the Worcester General Infirmary, on Monday, May 23, opened with a reference to the great loss sustained by the Empire in the death of Edward the Peacemaker, in a speech by the Chairman, who proposed that a resolution of condolence with the King and Royal Family be passed and sent to the King. This was seconded, and passed unanimously, the members all standing. The Finance Committee reported that the payment of the legacy of the late Mr. J. Corbett (£2,000) was expected shortly. The hon. medical staff recommended that in the place of the hon. anæsthetist (Mr. S. W. Coombs), who has consented to accept the post of hon. consulting anæsthetist, two hon. anæsthetists be appointed. They should attend in turn on operating days all emergency operations, and act respectively as medical and surgical pathologists. This recommendation, which was recognised as one conducing to the more efficient working of the institution, was carried. The hon. medical staff considered that some notice should be published in the local Press on the report of Sir H. Burdett. They considered that report favourable to the management of the infirmary, and would be glad to know what steps had been taken to give effect to the suggestions it contained. Dr. Crowe, in bringing forward the matter, proposed that a sub-committee be appointed to consider the whole subject thoroughly. The question had been raised some months ago, but so far no action had been taken. If a committee were appointed it could find out what was required to put the infirmary in a satisfactory and efficient condition, what the cost would be, and report to the Executive Committee. There was much discussion on this resolution, but it was recognised that the situation must be faced, and that the generous support which had enabled the infirmary to do so much good work in time past was not likely to fail in the present crisis. The motion was eventually carried and the sub-committee appointed. On the proposal to make a reply to Sir Henry Burdett's report, it was submitted that the points raised had been discussed by the House Committee both before and after the report had been made, and that no useful purpose would be served by making any further statement.

---

**Morpeth Cottage Hospital.**

Morpeth Cottage Hospital is fortunate in recording an extraordinary year—not only in regard to its expenditure. A ball, for instance, produced the very creditable sum of £74, and with smaller windfalls and subscriptions from other sources, not only were outstanding debts able to be paid, but the year was ended triumphantly with a balance of £1 7s. 8d. in the bank. The expenses were larger than before, but their patients were more numerous, sixty-nine instead of forty-six having been received. The accounts read as follows: income £408 15s., expenditure £407 7s. The patients themselves contributed on an average 7s. 6d. per week, the smallest sum received being half a crown, and the largest for the same period 15s. The district nurse went on many journeys, and made no fewer than 2,486 visits during the year. We believe that if the accounts of the various cottage hospitals noticed in this column were compared a high standard of prosperity would be found among them. The cottage hospital at Morpeth is no exception to this rule.

**St. Mary's Hospital.**

The chief new development perhaps disclosed in the recent annual report of St. Mary's Hospital, Paddington, was the opening last July of the wards on the first floor of the Clarence wing for patients undergoing treatment by inoculation. These wards, therefore, were in occupation for half the year, and allowed the patients two and a half times as long a period of treatment as those in other parts of the hospital, the average length of stay for inoculation patients being fifty-six days, as compared with twenty-two days for other patients. When the patients undergoing inoculation treatment are added to the other ward cases, a total of 4,242 in-patients were received during the year. The out-patient department treated nearly 3,000 more persons, 26,816 cases being entered there. It will be remembered that Sir Almroth Wright has had charge of the inoculation wards, which placed thirty-one beds at his disposal. Another change worthy of record was the conversion of the Boynton Ward into the *x*-ray department. This has been carried out ingeniously, the old ward being divided into two rooms, one for therapeutic, the other for screen work; the old bath room has become the dark room, and the lavatory a storage room for negatives. This department receives constant batches of patients from the skin department, and every available opportunity for the use of the *x*-ray treatment is bespoke for several weeks beforehand. According to the scheme for refurnishing some part of the hospital every year, the Lewis Lloyd Ward was refurbished; one of the seven already renovated under this scheme. St. Mary's has particular reason to complain of the scarcity of legacies, the debt on the year of £2,534 being attributed to this cause. The figures are: ordinary income, £19,475, plus legacies and £1,000 from the King's Fund, making a grand total of £25,914, and total expenditure, £28,449. The hospital is now in the serious position of owing an accumulated sum of £9,782. The Hospital Convalescent Home, Parkwood, Swanley, received 122 men and 79 women from St. Mary's, to the incalculable advantage of the patients, and to the hospital also, since the good work begun in the wards was completed at Swanley without any further expense to the London institution. Practically no case of abuse, we learn, was found in the out-patient department, a rare distinction of which St. Mary's may be proud, and to illustrate the force of this, only 15s. worth of bad debts has been recorded under the scheme by which surgical appliances are lent on the instalment system to out-patients and others who cannot afford to pay. Five members of the Medical School gained the M.D. of London University, and one of them the University Medal in Obstetrics. Another gained the M.S. of London, only two other candidates besides himself being successful. Indeed, the academic honours gained by St. Mary's Medical School are absolutely first rate—for instance, five candidates went up for the final F.R.C.S. examination, and every one of them was successful. During the year St. Mary's Medical School joined with the Middlesex Hospital Medical School in purchasing the freehold of an athletic ground at Park Royal, Acton. For details of individual interest our readers are referred to the columns of the *Hospital World*.

**St. Anne's, Soho, Choral Society at the London Temperance Hospital.**

An excellent concert was given on Saturday evening last by the above Society in the out-patients' hall in aid of the funds of the hospital. Mr. H. Goodey played the opening pianoforte solo, "Un fragment" (Mendelssohn), in a very able manner, after which the Society sang two madrigals, "Down in a flowery vale" (Festa), "Ah me! where is my true love?" (Anerio), and a part song, "Bells across the sea" (Macfarren). The renderings were enthusiastically received. Mr. Sydney Leader, the possessor of a beautiful tenor voice, sang "My Queen" in excellent style. The humorous part-songs of the Shaftesbury Glee Singers (Messrs. Wm. Gribble, J. Horncastle, T. Sweeney, and S. Gardner) were loudly encored, as was also Miss Gertrude Macaulay's beautiful singing of "Angus Macdonald." The old favourite "Chorus, Gentlemen!" was rendered in rollicking style by Mr. Lionel James, after which the Society sang three more delightful part-songs. Cowen's popular cantata "The Rose Maiden" constituted the second part of the programme. Miss Maud Birt, as Roseblossom, gave all the artistic touches necessary, in addition to displaying a beautiful soprano voice. Miss Gertrude Macaulay, as the gardener's daughter, scored a great success. Mr. James Horncastle gave a masterly reading of the Forester's part, and Mr. Stewart Gardner added to his many laurels as a baritone soloist. Excellent support was given at the piano by the Rev. G. W. Daisley, and the choruses were rendered with fine tone and precision.

